# SALON ontology for the formal description of sequence alignments

**DOI:** 10.1186/s12859-023-05190-7

**Published:** 2023-02-27

**Authors:** Antonio Benítez-Hidalgo, José F. Aldana-Montes, Ismael Navas-Delgado, María del Mar Roldán-García

**Affiliations:** 1 Departamento de Lenguajes y Ciencias de la Computación, University of Málaga, Málaga, Spain; 2 University of Málaga, ITIS Software, Ada Byron Research Building, Málaga, Spain; 3 Instituto de Investigación Biomédica de Málaga – IBIMA, Málaga, Spain

**Keywords:** Sequence alignment, Ontology, Linked data, Data integration, Semantic web

## Abstract

**Background:**

Information provided by high-throughput sequencing platforms allows the collection of content-rich data about biological sequences and their context. Sequence alignment is a bioinformatics approach to identifying regions of similarity in DNA, RNA, or protein sequences. However, there is no consensus about the specific common terminology and representation for sequence alignments. Thus, automatically linking the wide existing knowledge about the sequences with the alignments is challenging.

**Results:**

The Sequence Alignment Ontology (SALON) defines a helpful vocabulary for representing and semantically annotating pairwise and multiple sequence alignments. SALON is an OWL 2 ontology that supports automated reasoning for alignments validation and retrieving complementary information from public databases under the Open Linked Data approach. This will reduce the effort needed by scientists to interpret the sequence alignment results.

**Conclusions:**

SALON defines a full range of controlled terminology in the domain of sequence alignments. It can be used as a mediated schema to integrate data from different sources and validate acquired knowledge.

## Background

### Introduction

Sequence alignment is a well-known bioinformatics approach to identifying regions of similarity in DNA, RNA, or protein sequences. Finding the optimum alignment of two or more biological sequences allows for identifying highly-conserved regions resulting from functional, structural, or evolutionary relationships between the sequences. Thus, these techniques aim to compare nucleic sequences (DNA, RNA) or amino acid sequences (protein) across species or within a genome to establish regions of similarity. Software tools can produce these alignments in different formats, such as those generated by CLUSTAL W [1] or MAFFT [2], making comparative analysis a complex task. Thus, providing a unified way of representing sequence alignments would help developers compare different tools. Moreover, a unified representation would ease the interpretation of results from different sequence alignment tools. Semantic Web technologies have been used in the past to represent biological data [3] to unify their representation and enable the integration of these data.

Scientific workflows may use the sequence alignment results as inputs for other analyses. For example, several computational biology techniques, such as sequence homology [4] and phylogenetic analysis [5], rely on pairwise or multiple sequence alignments. This fact makes the quality of sequence alignment algorithms (correctness of these alignments) a vital issue in more complex studies. Thus, automatically validating the sequence alignments is a key task for these computational biology techniques. The Semantic Web community has developed a series of standards, such as the Web Ontology Language (OWL) [6] and the Semantic Web Rule Language (SWRL) [7] for the formal representation of information enabling mechanisms to validate its correctness. OWL 2 is the latest version of the W3C standard ontology language and is the primary language for biomedical ontologies nowadays. SWRL provides the OWL ontologies with procedural knowledge compensating for some limitations of ontology inference, particularly in identifying semantic relationships between individuals.

Moreover, with the development of high-throughput platforms, a boundless amount of biological data is available, which comprises the sequences themselves (i.e., using high-throughput genome sequencing techniques) and other biologically relevant information. This information may include the name of the species (organism), a brief description of the sequence and the sequence type (i.e., nucleotide or protein sequence). Some other useful information can be derived from the former, such as metrics about the alignment quality using score functions. Integrating such information with the sequence alignment information would help researchers find relevant data and enhance the quality of the interpretation of the retrieved results. The enormous potential of ontologies allows a wide range of applications in integrative research. In this sense, ontologies play a crucial role in data integration and data management in the context of life sciences and biomedical research. Linked Open Data Cloud [8] (LOD Cloud) is an effort started in 2007 to collect information on the existing Linked Open Data repositories and how they are connected. This classification effort has grown from the initial 12 datasets to the 1,301 datasets (with 16,283 links) listed in May 2021. An important part of the registered repositories is classified under the Life Sciences domain (around 30% of the datasets with the domain annotated correspond to Life Sciences, 23% of the whole LOD Cloud). Thus, LOD Cloud can serve as a way to retrieve relevant information in this domain.

The FAIR Guiding Principles [9, 10] aim at improving the Findability, Accessibility, Interoperability, and Reuse of digital assets. These principles are being adopted in life sciences to enable machine-actionability of the biological data to deal with the increasing volume, complexity, and creation speed of data. While Linked Open Data is more focused on data interoperability, FAIR principles are more oriented to data reuse. However, they have similar approaches [11]. Thus, this work aims to apply both approaches to improve the quality of the results.

This paper presents SALON, a novel OWL 2 ontology to formally represent pairwise and multiple-sequence alignments. Consequently, there is native support for reasoning and consistency checking on this ontology. This ontology provides classes and properties to formally describe the former domain, including alignment construction methods and scores to assess its quality. This ontology is complemented with a set of SWRL rules to enhance the validation capabilities. This study presents an ontological approach to annotating data before its consolidation and enrichment from different Linked Open Data sources. As a proof of concept, we have used UniProtKB Linked Data Endpoint to retrieve information on aligned proteins. Thus, the SALON ontology can be used as a mediated schema integrating alignments with protein sequences. The generated RDF can be stored in a single RDF repository with formal reasoning support or even used in Open Source tools such as Protègè [12]. SALON ontology complies with current best practises for ontology development and publication, and FAIR principles. As a result, we use globally unique, persistent and resolvable identifiers. To illustrate the usefulness of our proposal, we include three compelling use cases in bioinformatics, where SALON is the core schema for data integration and exploitation.

### Prior work

Thompson et al. [13] describe a Multiple Alignment Ontology (MAO), an OBO ontology for data retrieval and exchange in the fields of multiple sequence alignments (DNA/RNA/protein), and 3D structures. The MAO ontology contains multiple cross-references to related ontologies and other datasets, e.g., UniProtKB [14] and Genbank [15]. It provides a task-oriented design to facilitate communication between different methods for constructing, analysing and annotating sequence alignments. To this end, the top-level concept of MAO, namely *multiple_sequence_alignment*, defines a subset of sequences, which in turn defines alignment sequences and columns. Hierarchical relationships between concepts are defined by *is_a* (specialisation relation) and *part_of* (partitive relation). Each of these concepts can then be characterised according to attributes. For example, MAO defines an associative relation, *is_name*, which allows specifying a user-defined name for a given sequence to provide a further understanding of such sequence. It also allows the integration of 3D structural information about proteins. However, as of 2015, the ontology is tagged as deprecated/inactive in the OBO Foundry website [16].

Furthermore, the MAO ontology is only available in OBO format, which can not take advantage of automated reasoning for ontology validation and the search for subsumption hierarchies. However, it can be translated to OWL (as OBO can be considered a subset of OWL). Due to the nature of OBO, most knowledge is represented as labels, lacking OWL entailments related to reasoning. Therefore, this ontology is a simple approach to representing this kind of knowledge, limiting its use to the presentation, interoperability, and data sharing between multiple alignment protocols.

KinView [17] is a visual comparative sequence analysis tool. It includes an ontology MSAOnt, a simple schema for relating sequences to a profile or consensus sequence. MSAOnt is compared to MAO, asserting that MSAOnt is a minimalist representation of a profile or consensus alignment components, while MAO is a more generic ontology. The paper does not provide details about the ontology, and it appears as not available for download.

This paper presents an approach that reuses MAO conceptualisation and extends it with additional OWL, mainly object and datatype properties and SWRL axioms, to enable data validation.

## Methods

The proposed ontology has been developed using the standard ontology 101 development process [18], consisting of seven steps. Below we describe how these steps have been approached in this work:

1. *Determine the domain and scope of the ontology* SALON is focused on the description and formalisation of biological sequence alignments.
2. *Consider reusing existing ontologies* We have reviewed the OBO Foundry ontologies seeking existing ontologies that match SALON scope and intended application. We did not find any other related ontology apart from MAO.
3. *Enumerate important terms in the ontology* Essential terms in the ontology are those needed to describe biological sequence alignments. These terms have been extracted from MAO and manually extended with other concepts defined, taking information from the scientific literature in the field. Examples of such terms are Alignment, Alignment Sequence, Alignment Column, Feature, and Sub Alignment. Furthermore, terms for describing alignment construction methods are selected, for example, Deterministic Approach and Stochastic Approach. Finally, we included terms regarding scores, such as Alignment Score, Column Score, and Column Score Function.
4. *Define classes and the class hierarchy* Classes in the ontology correspond to important terms. In contrast with MAO, complete covering of 3D structural information is not included in SALON. We are focused on merely describing sequence alignments, ranging from a single residue to a set of sequences (i.e., multiple sequence alignment) and its construction method/scoring. Examples of classes in the ontology are: *Alignment*, *AlignmentSequence*, *AlignmentColumn*, *ConstructionMethod* and *AlignmentScore*. We have defined *DNAAlignmentSequence* and *ProteinAlignmentSequence* as subclasses of *AlignmentSequence*. *DeterministicApproach* and *StochasticApproach* have been defined as subclasses of *ConstructionMethod*.
5. *Define the properties of classes and slots* Object properties and data properties (slots) are defined to describe Alignments. For example, an alignment has columns and contains sub-alignments. A sub-alignment has sequences, an alignment sequence has features, an alignment has a score, and a sub-alignment has a construction method. On the other hand, alignment sequences are described by the data properties character, identifier, accession number, keyword, and score value.
6. *Define the facets of the slots* This step includes the definition of cardinality constraints and value restrictions. An example of cardinality constraint in our ontology is that a Sub-alignment has at least two Alignment sequences.
7. *Create instances* Ontology Individuals are obtained by mapping the data from different data sources to RDF according to the ontology specification. One of the data sources used in the proposed use cases is the benchmark alignment database BAliBASE [19].

The SALON ontology has been developed using the Prótegé OWL editor version 5 in OWL 2 format. To assess the compliance of the ontology against the FAIR principles, we have used the FOOPS! validator [20], a web service for detecting best practices according to each FAIR principle, scoring the highest mark in 19 out of 24 tests.

### SALON Concepts and relationships

**Fig. 1:**
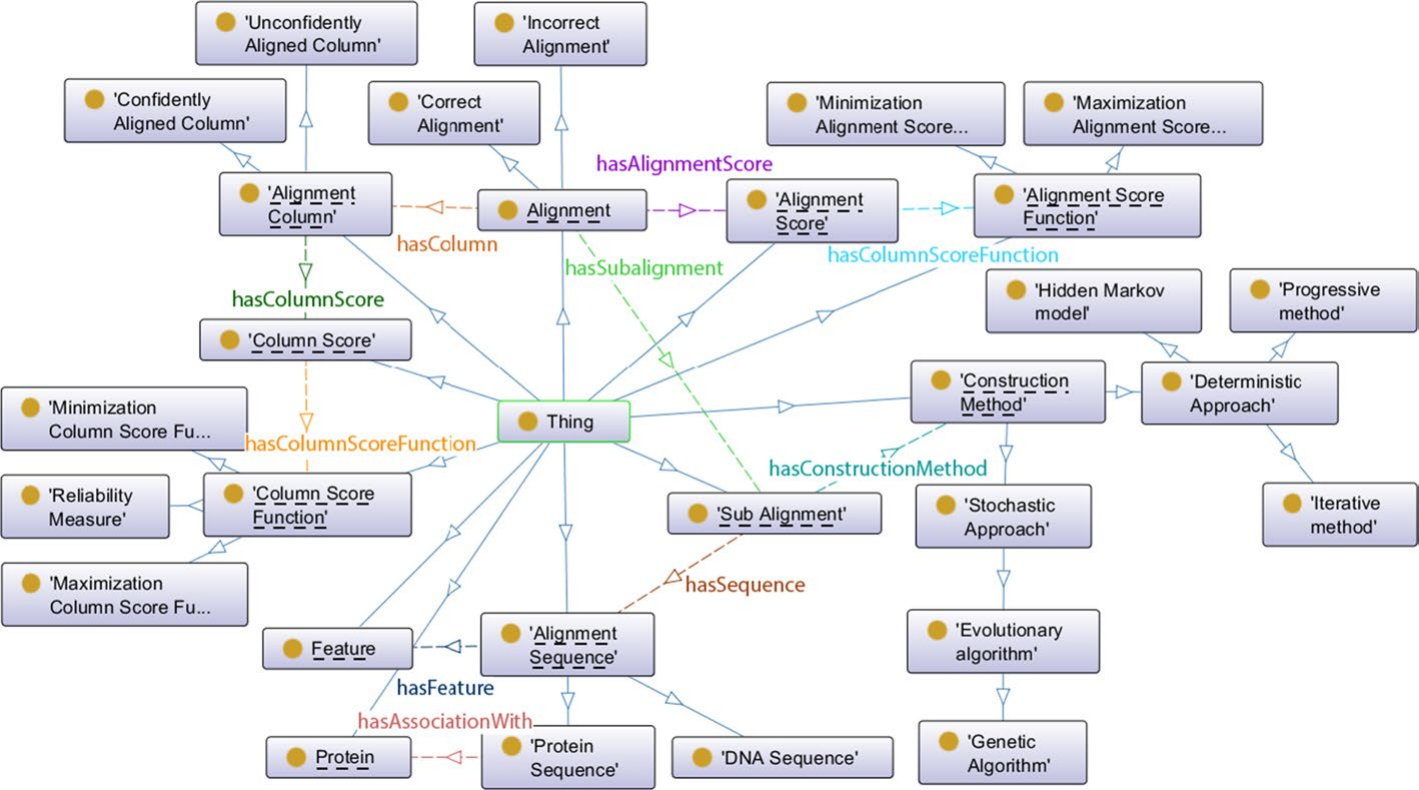
Classes in SALON. Underline classes represent top-level classes in the ontology. The *is-a* relationship is depicted as blue arrows, with object properties depicted as dashed arrows. Boxes represent classes

The proposed ontology available at https://w3id.org/salon consists of 30 classes, 14 object properties (see Table 1) including 4 inverse object properties to ensure linking to related resources, 27 data properties and 144 logical axioms. The top-level classes of the ontology are *Alignment*, *AlignmentColumn*, *AlignmentScore*, *AlignmentScoreFunction*, *AlignmentSequence*, *ColumnScore*, *ColumnScoreFunction*, *ConstructionMethod*, *Feature*, *Protein*, and *SubAlignment* (see Fig. 1).

The *Alignment* class represents a pairwise or multiple sequence alignment. An *Alignment* contains at least one *SubAlignment*, representing a subset of sequences with some property in common. This can be useful to represent clusters of sequences within the alignment, e.g., phylogenetic trees for multiple sequence alignments.

Attributes of *AlignmentColumn* are *ColumnScore*, *ColumnWeight*, *ConsensusCharacter*, among others. Some attributes of *AlignmentSequence* are *organism* or *keyword*. Table 2 shows SALON data properties. Range in data properties correspond to a datatype, i.e. string, integer, etc.

**Table 1:** SALON object properties

Object property	Domain	Range	Inverse
hasConstructionMethod	SubAlignment	ConstructionMethod	–
hasAlignmentScore	Alignment	AlignmentScore	–
hasAlignmentScoreFunction	AlignmentScore	AlignmentScoreFunction	–
hasColumn	Alignment	AlignmentColumn	isColumnOf
hasColumnScore	AlignmentColumn	ColumnScore	–
hasColumnScoreFunction	ColumnScore	ColumnScoreFunction	–
hasSubAlignment	Alignment	SubAlignment	isSubalignmentOf
hasFeature	AlignmentSequence	Feature	isFeatureOf
hasSequence	SubAlignment	AlignmentSequence	isSequenceOf
hasAssociationWith	ProteinSequenceAlignment	Protein	–

**Table 2:** Part of SALON data properties

Data property	Domain	Range
atSequenceIndex	AlignmentColumn	Integer
column	AlignmentColumn	String
score	AlignmentScore or ColumnScore	Integer or float
scoreMax	AlignmentScore or ColumnScoreFunction	Integer or float
scoreMin	AlignmentScore or ColumnScoreFunction	Integer or float
scoreCutoff	ReliabilityMeasure	Integer or float
FScore	Feature	Integer or float
columnWeight	AlignmentColumn	Integer
consensusCharacter	AlignmentColumn	Short
gapCharacter	SubAlignment	Short
accessionNumber	AlignmentSequence or Protein	String
identifier	AlignmentSequence	String
length	AlignmentSequence	Integer
organism	AlignmentSequence	String

### SALON instance generation

This section focuses on the automatic generation of SALON instances from sequence alignment data. This transformation process is done from an input file in MACSIM XML format [21], and produces the corresponding RDF triples (SALON instances) as seen in Table 3. To this end, we have developed a Python client package available at GitHub [22] with several utilities to work alongside an RDF repository. For RDF manipulation, we use the rdflib library [23], which generates an RDF graph that can be serialised into a plethora of semantic-aware formats such as RDF/XML, NTriples, Turtle, and JSON-LD. The RDF file can be loaded in the Prótegé OWL editor to visually inspect the sequence alignment data. It is worth noting that new mapping functions can be applied to alignments in other file formats, producing SALON instances.

**Table 3:** XML mappings from MACSIMXML to SALON classes. Some SALON data properties, such as *gapCharacter*, are derived from the sequence itself and are not shown in the table

XML	Direct mappings
|<aln-name></aln-name>|	salon:SubAlignment class, salon:subAlignmentName
|<sequence seq-type=“Protein”></sequence>|	salon:ProteinAlignmentSequence class
|<seq-name></seq-name>|	Salon:identifier
|<accession></accession>|	salon:accessionNumber
|<sequence></sequence>|	salon:sequence
|<fitem></fitem>|	salon:hasFeature
|<ftype></ftype>|	salon:FType
|<fstart></fstart>|	Salon:FStart
|<fstop></fstop>|	salon:FStop
|<fnote></fnote>|	salon:FNote

### Ensuring alignment correctness

Scoring methods for sequence alignments assign numerical values to differentiate good alignments from poor ones. The alignment score in pairwise alignments is computed based on matches/mismatches and gaps in both sequences. In multiple alignments, scores can be determined based on scoring functions. The score function is independently computed for every column in the alignment. The alignment score will be the sum of these values. It is important to note that there is no consensus about the best metric to score a multiple sequence alignment, so several functions exist to this end.

Some examples of score functions include the percentage of totally conserved columns, percentage of non-gaps, and entropy [24] scores. For example, the percentage of totally conserved columns and percentage of non-gaps scores can be either 0 or 1 for a given column (i.e., *ColumnScoreFunction*), and ranges from 0 to 100 (representing a percentage) for the whole alignment (*AlignmentScoreFunction*). The entropy value of any given column in a multiple sequence alignment ranges from 0 (only one residue is represented in that position of the sequence) to 4.322 (all 20 residues are represented). Thus the alignment scores a maximum of $\text {number of sequences} * 4.322$. On the other hand, reliability score functions such as GUIDANCE [25] and Heads-or-Tails [26] help us quantify the robustness of the sequence alignment. For example, GUIDANCE assigns a confidence score between 0 and 1 for each column in the alignment, which can be used to filter unreliably aligned regions before subsequent analysis.

SWRL is a rule language that incorporates mechanisms to identify semantic relationships between individuals [7], hence providing OWL-based ontologies with extra inference capabilities. SWRL is based on rule expressions in the form of “Antecedent $\Rightarrow$ Consequent” to represent semantic relationships. SALON includes several SWRL rules to determine if any score value is out of range based on the underlying score function, which typically fits into an upper and lower bound. These rules can help scientists to ensure alignment correctness automatically.

**Fig. 2:**
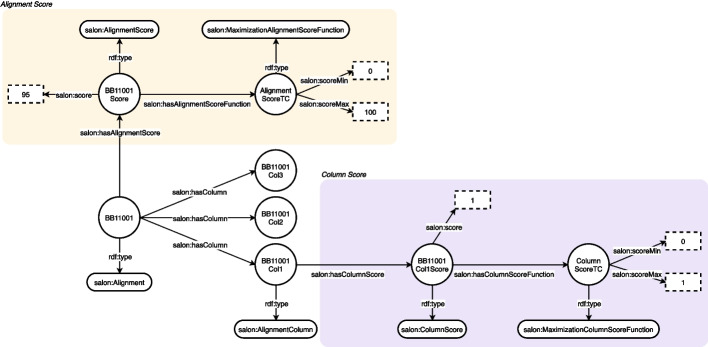
Alignment and column score functions represented according to SALON. The *Alignment* contains one *AlignmentScore* which scores 95. The underlying *MaximizationAlignmentScoreFunction* ranges from 0 to 100. Additionally, one *ColumnScore* scoring 1 is included in the figure. It contains one *MaximizationColumnScoreFunction* ranging from 0 to 1

For example, to check if the alignment in Fig. 2 is correct, we have defined the SWRL rule in Listing 1. This rule is generic and is valid to check the correctness of any alignment modelled according to SALON. To check if an alignment is incorrect, we have defined four SWRL rules to consider all the incorrectness possibilities, i.e. $column score > column scoreMax$, $column score < column scoreMin$, $alignment score > alignment scoreMax$ and $alignment score < alignment scoreMin$. Listing 2 shows the rule to check the latest case. The SWRL rule in Listing 3 checks for unreliable columns in any alignment modelled in SALON according to a reliability score function.

SWRL rules require a reasoner that supports them. The rules engine uses alignments and rules as inputs for inferring new facts, e.g., if the alignment is correct or not. Furthermore, as SWRL rules are built in the ontology, no external validation system is required to ensure alignment correctness.

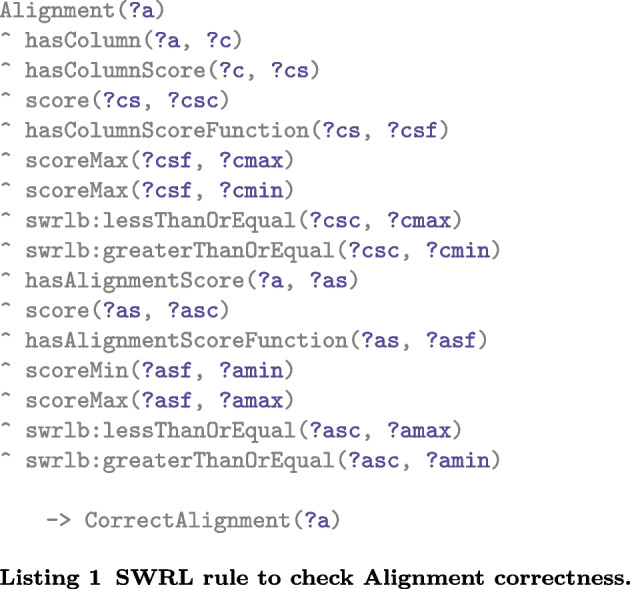

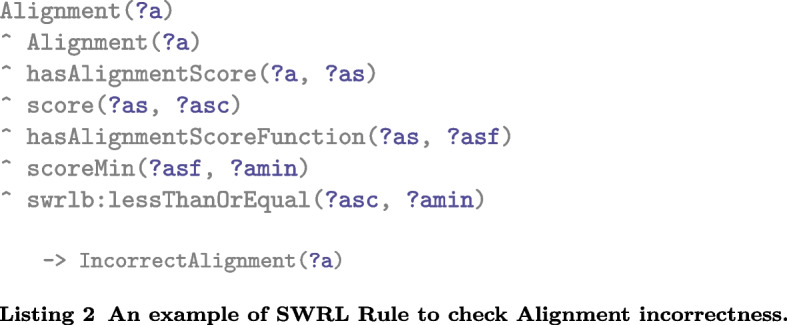

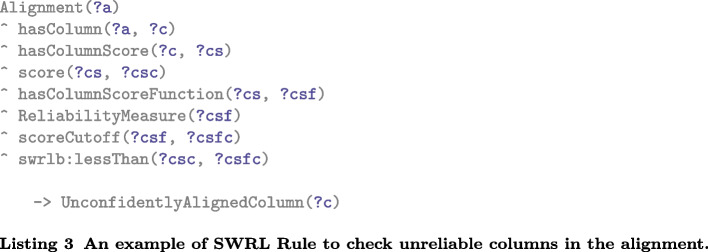







### RDF repository

To test how the ontology instances can be used in real scenarios, we have deployed an on-premises instance of Stardog Knowledge-graph platform. Stardog supports the SPARQL query language and thus provides access to the data via the SPARQL protocol, a declarative language for performing operations over SPARQL endpoints, capable of receiving and processing those requests. Unlike other triple stores, Stardog supports SWRL rules (which this ontology uses). Therefore, SALON requires a running Stardog server instance to unlock all features at the server side (to provide additional functionalities such as testing user interfaces on top of it). Virtuoso or any other triple store can be used, but in such a case, SWRL will not be evaluated, and the validation will be limited to the inference support provided.

RDF instance files can be inserted into Stardog using a SPARQL update query. Automatic scripts for RDF repository population using Python can be found at GitHub [22].

### Semantic enrichment of protein sequences

Due to the increasing use and development of high-throughput platforms, many biological data are available in public databases and repositories. Usually, databases provide different information regarding the same biological structure. Bringing together all this information into a single and unified repository might be very time-consuming. SALON can help to address this issue. The SPARQL protocol defines a federated form of the SPARQL query language that provides access to remote endpoints. Those queries (which can be issued to a SPARQL endpoint) are not constrained to working with a single database, but federated queries are supported across multiple endpoints. This powerful feature enables the online query of several related SPARQL Endpoints. However, the resolution of such queries relies on the availability of the individual SPARQL Endpoints to provide a complete answer.

This way, complete information from several Open Linked Data resources can be integrated using a single query through the federation. For example, we can use the information already stored in our repository about protein sequences to integrate data from other data sources. When a new alignment record is inserted in our RDF repository, a federated query is automatically triggered against UniProt Knowledgebase (UniProtKB), the most well-known databases of protein structures and functional information. UniProtKB stores data about proteins, e.g., source organism, name, NCBI identifier, and gene, and provides a public accessible SPARQL endpoint [27].

However, the Linked Data sources’ distributed nature makes these federated queries fail due to the system failure in one of its parts. For this reason, in the example described in this section, the additional information returned with the federated query is inserted into the local database.

The sequence’s accession number can be used, for example, to retrieve 3D protein structures and compare alternative multiple sequence alignments in terms of their relative accuracy [28]. SALON can use this identifier (represented by the *accessionNumber* data property) to find matched records from UniProtKB and retrieve information that would aid subsequent analyses. Listing 4 shows the corresponding federated SPARQL query to aggregate information about the protein 1D2N. The range of the SALON *accessionNumber* data property is a string containing the UniProtKB entry of the protein. Using this value, we obtain all the information related to such a protein and insert it in our RDF repository.
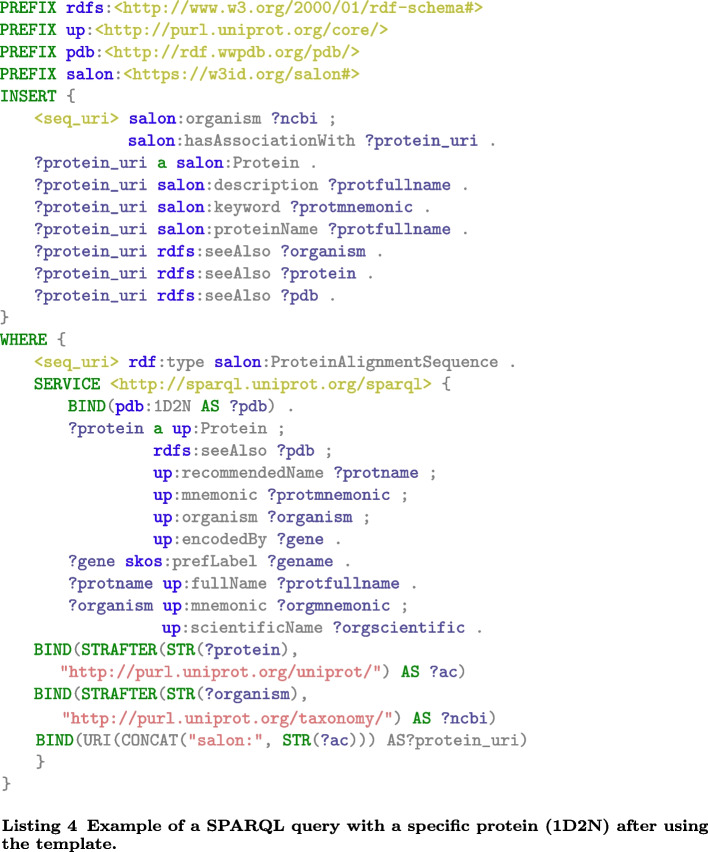


Note that this information about each protein involved in the alignment process is unavailable in the alignment data. Thus, a user obtaining the alignment will need to retrieve such information from UniProtKB. Traditionally this could be done by manually exploring the UniProt portal with a vast human effort (for alignments with many proteins). An alternative would be to develop a software application using the UniProt services to retrieve such data, with the required programming skills from the user. Federated SPARQL queries simplify this process to retrieve updated information related to this data.

### Generation of FASTA description lines

The FASTA format is used to represent biological sequences. Still, it lacks a strict standard specification, translating into consistency issues as bioinformatics tools fail to parse description lines correctly. This problem can cause malfunctioning of third-party tools and unexpected results when parsing those files. There are cases in which FASTA files’ description lines are poorly described, or some information about the sequences (such as their source organism or accession number in reference databases) is missing.

**Fig. 3:**
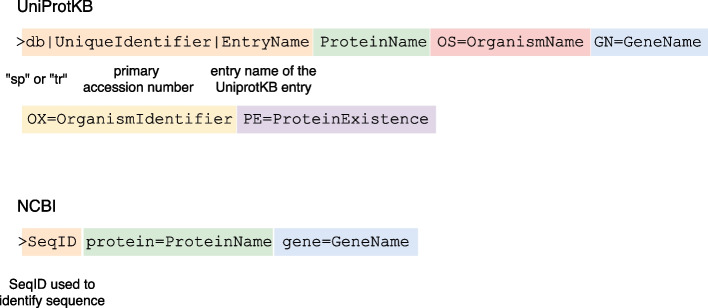
Formatting of FASTA header/description line (including optional modifiers) for a protein sequence in UnitprotKB and NCBI. For the sake of simplicity, some modifiers have been omitted

**Fig. 4:**
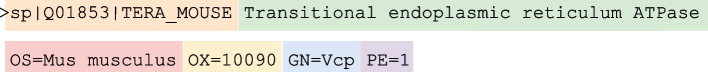
UniProtKB FASTA description line for a protein sequence with known PBD identifier

For example, UniProtKB and NCBI provide different specifications about the required and optional information in their sequences’ header lines (see Fig. 3). It can be very time-consuming to manually retrieve missing tags from different sources to switch between specifications. Thus, a mapping must be applied between UniProtKB and NCBI resources to find cross-referenced entries. SALON can help scientists overcome this issue by filling the information gaps between such services. As mentioned above, the proposed RDF repository integrates information about sequences from different repositories, which is stored after a new alignment record is inserted by running a federated SPARQL query. Therefore, a specific SPARQL query can be run to obtain the desired information and compose a custom description line for the desired service. For example, the SPARQL query presented in Listing 5 can be used to obtain the different fields needed to build the header in the UniProtKB format: i.e., db, UniqueIndentifier, EntryName, ProteinName, OrganismName, GeneName, and ProteinExistence (see Fig. 3), when the only known information is the PDB identifier of the associated protein. Figure 4 describes the header generated. Without the SALON RDF repository, the scientist would have needed to search in the reference database for the entry (or entries) that matches the PDB identifier of the related protein. It would then have been required to manually extract all the required information to construct the description line (or description lines, if a PDB identifier matches several entries). The sequences/alignments can be meaningfully compared and integrated using SALON to standardise FASTA file headers. Informative labels can be used in, for example, phylogenetic analysis, where the taxonomic lineage can be used to evaluate the phylogenetic tree.
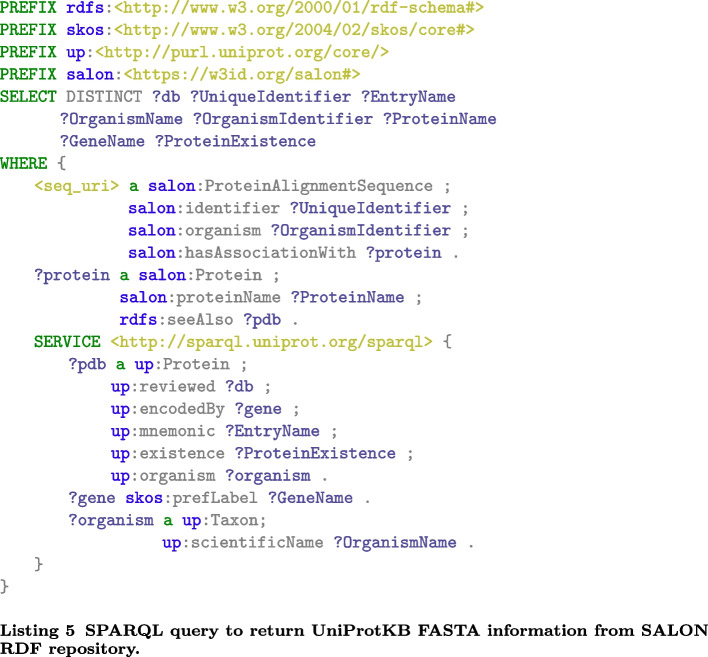


It is frequent that, when comparing a highly conserved protein or domain of such a protein from two closely related species, a single PDB identifier can be mapped to several UniProtKB entries. In those cases, manual intervention by the domain expert might be required to help identify the most suitable option for the experiment required.

## Results

To test SALON, we have focused on a set of manually constructed and verified alignments from a relevant dataset such as BAliBASE 4.0. This dataset provides high-quality reference alignments of DNA and protein sequences in XML and FASTA formats. In this database, each multiple alignment contains information about the sequences and some quality measures related to the alignments themselves, such as the column score, i.e., the fraction of aligned columns that are correctly reproduced.

**Fig. 5:**
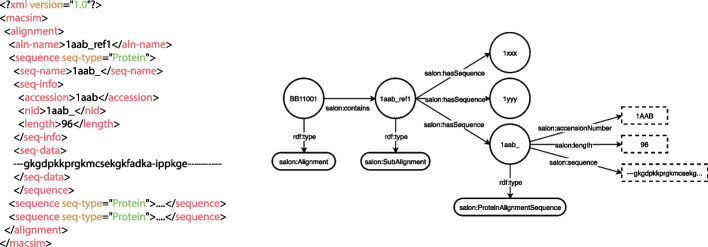
BB11001 alignment from BAliBASE (left) represented in RDF according to SALON (right). In this example, our multiple sequence alignment contains a sub-alignment of three protein sequences of known name, accession number, length and primary structure. Only one of the three sequences (bottom right corner) is fully represented for the sake of clarity

Figure 5 depicts a simplified representation of a multiple sequence alignment as an RDF Graph using SALON Schema. On the left side, an alignment in MACSIM XML format is partially represented (i.e., BB11001 instance from BAliBASE). On the right side, the same alignment is shown according to our proposal, where nodes correspond to subjects or objects, and edges correspond to predicates (according to the RDF terminology).

**Fig. 6:**
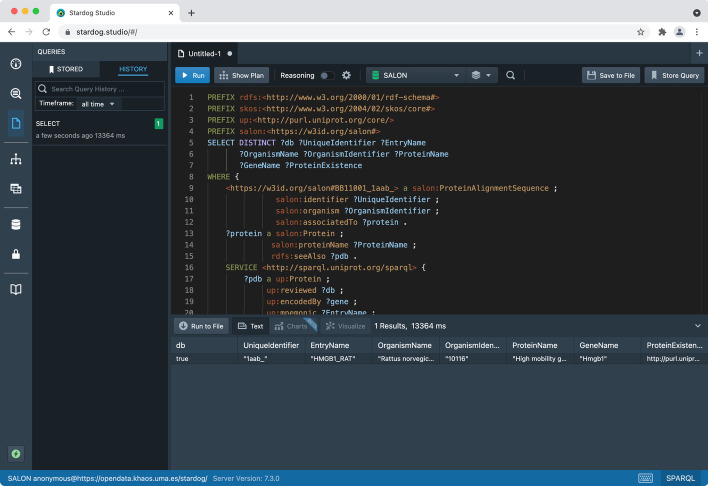
Sample query in Stardog Studio. This query returns additional information associated with the sequence *salon:BB11001_1aab_* from UniProtKB database

**Fig. 7:**
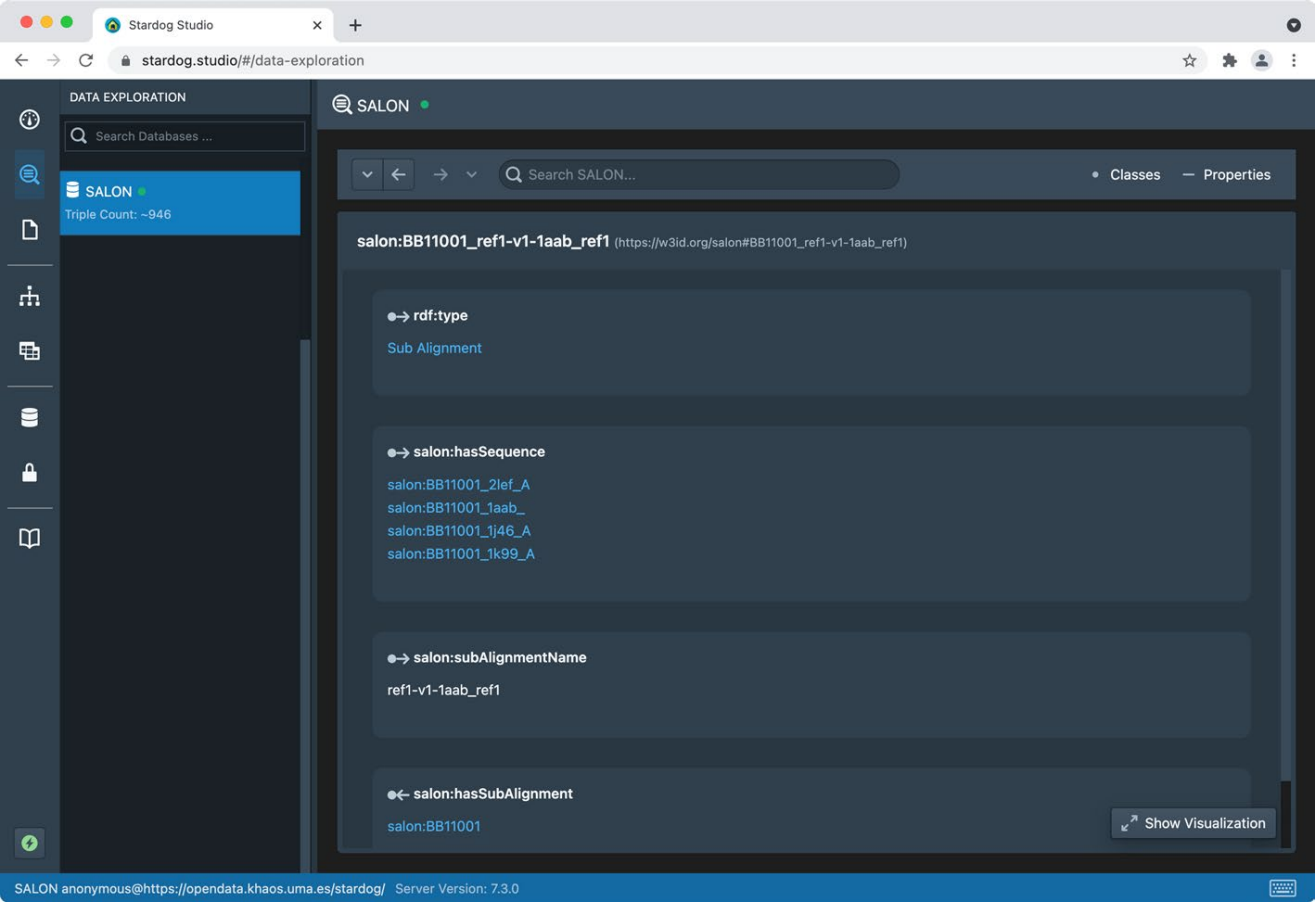
Stardog Studio Data Exploration tab showing an alignment represented using SALON (i.e., BB11001 instance from BAliBASE). This sub-alignment contains four sequences, namely *2lef_A*, *1aab_*, *1j46_A*, and *1k99_A*

BAliBASE alignments can also be downloaded in FASTA, a text-based file format representing either nucleotide sequences or peptide sequences using single-letter codes. FASTA files contain headers or description lines, distinguished from the sequence itself by a greater-than symbol. The information in the description line of the sequence, e.g., its protein identifier or source organism, can also be mapped to their corresponding terms in the ontology using a custom mapping function to transform FASTA to semantic-aware formats, i.e., RDF. When not present, this data can be retrieved from different sources to construct the description line. By matching identifiers found in the description line of a sequence against entries in authoritative biological databases, we can add informative labels to ensure that third-party tools can be used without adaptation. This consistency problem has been addressed in the past by web tools such as SeqScrub [29], but no SPARQL-based approach has been applied to the best of our knowledge. Figure 6 shows the SPARQL query in Listing 5 using Stardog Studio [30], a free web-based tool for working with Stardog RDF repositories. This query retrieves the missing labels needed to construct the description line of a sequence by querying UniProtKB, obtaining the gene name and the organism related to a given protein, among others. Then, the FASTA file header is constructed as shown in Fig. 4. The SALON ontology instances for these data are then stored in the RDF repository to enhance sequence alignment information. To explore these results, a SPARQL endpoint is available at [31]^1^. Stardog Studio also provides a SPARQL query editor for exploring the alignment data, as shown in Fig. 7.

**Fig. 8:**
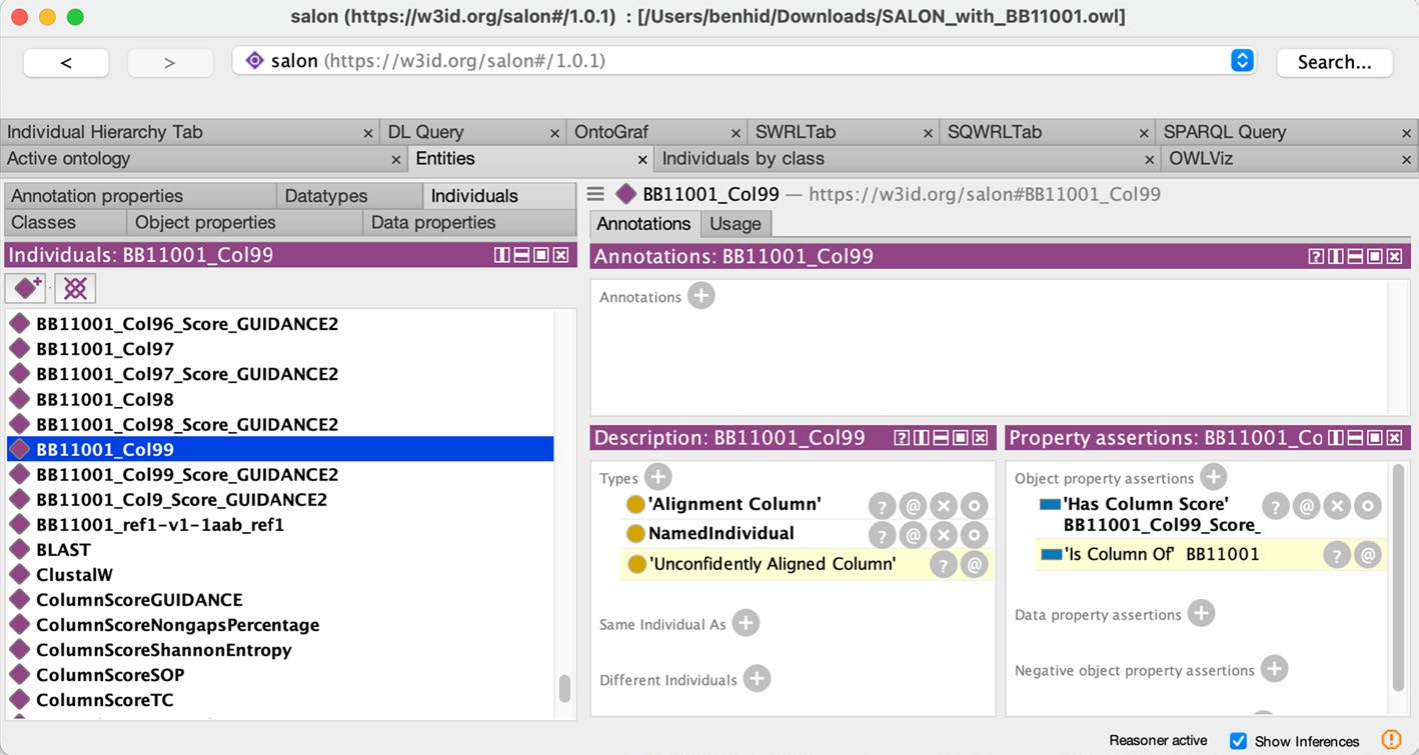
Protégé reasoning engine can be used to infer rule engine knowledge. In this example, the rule engine shows unreliable columns in the BB11001 alignment

Additionally, these instances are provided in the GIT repository so users can use any Open Source tool with RDF and SWRL support (such as Protègè). With the incorporation of SWRL rules, alignments can be validated to ensure their correctness and robustness automatically. For example, an alignment inferred as incorrect by the reasoner can be due to an error in the underlying alignment algorithm, which can dramatically impact subsequent steps in the pipeline. Similarly, Fig. 8 shows one column in the alignment inferred as unconfidently aligned by the reasoner. A further inspection of the Protégé Editor shows that, according to our data, the GUIDANCE confidence level of that column exceeds the cutoff and thus is marked as unreliable. We can remove unconfidently aligned columns to reduce errors caused by alignment errors in subsequent analysis [32, 33].

## Conclusions

The alignment of biological sequences is probably one of the most critical tasks in bioinformatics. Sequence alignments have many applications, such as gene finding, gene function prediction, or genome sequence assembly. Therefore, the availability of biological databases with information about correct and complete sequence alignments is of particular interest to scientists and researchers in this area. Several studies show that biological knowledge is growing and is usually distributed among many databases. Querying specific knowledge about proteins, genes, etc., can improve a sequence alignment by enriching it with relevant biological information. Efficient data integration is a challenge in general, and it is a bottleneck for biological sequence alignments enrichment. Semantic Web technologies simplify access to heterogeneous biological databases through standard models of knowledge representation.

Ontologies are considered a central part of the Semantic Web for the formal description and representation of domain knowledge. Annotating biological sequence alignment (and its associated information) with an ontology allows the development of linked data repositories, providing homogeneous access to heterogeneous information and enabling intelligent and advanced applications. In this direction, we presented SALON, an ontology describing pairwise and multiple sequence alignments. Our proposal defines a valuable and comprehensive vocabulary for the representation of biological sequences using a full range of controlled terminology in the context of sequence alignment. Its usability has been proved constructing an RDF repository with information on sequence alignments. The algorithms developed to populate this repository from existing datasets are also provided so scientists can replicate this process for their data at [22].

The SALON ontology can also play as a mediated schema for the virtual integration of data from different sources using federated SPARQL queries. These data can then be gathered to, for example, generate description lines of FASTA sequences or exchange header line formats between different services. To validate sequence alignments, SALON can be further exploited by defining SWRL rules, which automatically determine if a sequence alignment is plausible based on its global assigned score. This feature has been tested in the BAliBASE dataset but can be applied according to the scientists’ data.

In future work, we plan to use these results to create a novel database on sequence alignments to enable an easy way to use this software solution by users with limited technical knowledge. This includes defining new classes and properties to describe sequence alignments’ construction methods in collaboration with domain experts. In the near future, we plan to submit our proposal to the Linked Open Vocabularies (LOV) catalogue, which, in addition to Zenodo, is aimed to encourage the use of the ontology by practitioners and other scientists in the field.

## Data Availability

The proposed ontology is available at https://w3id.org/salon, and the repository archive is accessible via Zenodo at 10.5281/zenodo.5506946.

## Abbreviations

OWL: Web Ontology Language
RDF: Resource Description Framework
OBO: Open Biological and Biomedical Ontologies
SPARQL: SPARQL Protocol and RDF Query Language
SWRL: Semantic Web Rule Language
XML: Extensible Markup Language
PDB: Protein Data Bank

## Footnotes

1 Authentication credentials *salondemo*:*salondemo*.
